# PP47 The Role Of Target Product Profiles Within Health Technology Assessment: A Narrative Review And Case Study

**DOI:** 10.1017/S0266462324002186

**Published:** 2025-01-07

**Authors:** Gareth Hopkin, Anastasia Chalkidou, Farhan Ismail, Charlotte Pelekanou, Mark Salmon, Dalia Dawoud

## Abstract

**Introduction:**

Target product profiles (TPPs) can be used to outline the minimal and desirable characteristics of a health technology to meet a particular health need. They have been used by industry, regulators, and international health agencies to guide product development and facilitate communication across stakeholders, but there appears to have been limited consideration of their utility within HTA.

**Methods:**

We completed a narrative review of academic and grey literature on uses of TPPs across the product development, regulatory, and HTA pathway and considered areas within HTA where TPPs could have a role. We then developed a case study alongside an assessment of virtual ward platforms for acute respiratory infection. This case study aimed to explore whether it was possible to construct a TPP to support guidance based on HTA committee discussions and to consider associated opportunities and challenges. We also compared our TPP with other product specifications used within NHS England to support commissioning.

**Results:**

There has been discussion about how industry can build HTA perspectives into TPPs. However, we did not identify use of TPPs led by HTA agencies. Two use cases of TPPs were identified: (i) to support demand signaling for areas of unmet need and (ii) to provide a framework for class-based recommendations. HTA committee discussions supported the development of a TPP for virtual ward platforms with domains relating to patient population, technological specification, evidence requirements, and other factors (e.g., patient choice). Challenges around the time needed to consider both evidence and desired attributes and overlap with approaches used in commissioning were identified.

**Conclusions:**

TPPs may have a role to play within HTA to highlight areas of unmet need or to support innovative approaches to guidance. Digital health technologies may be particularly amenable to these approaches due to the larger range of similar products and ease of replicability. Further work should define how TPPs can be used to their full potential.

